# Longitudinal Patterns of Racial Discrimination in Midlife and Older Black Adults and Implications for Healthy Aging

**DOI:** 10.1093/geroni/igaf122.1690

**Published:** 2025-12-31

**Authors:** Kellee White Whilby, Shuo Huang, Bethany Bell

**Affiliations:** University of Maryland College Park, College Park, Maryland, United States; University of Maryland Institute for Health Computing, Baltimore, Maryland, United States; University of Virginia, Charlottesville, Virginia, United States

## Abstract

Single cross-sectional assessments of racial discrimination may overlook its dynamic, cumulative patterns. Capturing these trajectories could provide deeper insight into age-related health risks and resilience. However, few studies have examined racial discrimination trajectories, especially among midlife and older Black adults. This study characterizes racial discrimination trajectories and identified correlates of the trajectories among a sample of middle-aged and older black adults over a 12-years. Using data from the Health and Retirement Study (2006–2020, N = 1,710, 50+), repeated measures latent profile analysis was employed to identify racial discrimination trajectories which were constructed based on the Everyday Discrimination Scale. Multinomial logistic regression models were used to identify predictors of membership for each racial discrimination trajectory class adjusting for sociodemographic, health behaviors, social and psychological resilience characteristics at baseline. Three racial discrimination trajectories were identified: low-stable (70%), moderate (23%), and persistently high and increasing (7%). Individuals reporting higher levels of major lifetime experiences of discrimination and greater neighborhood social cohesion were associated with membership in the “moderate” and the “persistently high and increasing” racial discrimination trajectory groups. Those reporting positive social support and psychological resilience were less likely to be in the “moderate” or the “persistently high and increasing trajectory” groups. These findings suggest variability in the cumulative patterning of racial discrimination among midlife and older Black adults. Discrimination trajectories may offer more precise estimates of the health consequences of cumulative exposure. Future studies should explore whether membership in specific discrimination trajectory groups confers differential risk for age-related conditions.

